# A little damping goes a long way: a simulation study of how damping influences task-level stability in running

**DOI:** 10.1098/rsbl.2020.0467

**Published:** 2020-09-23

**Authors:** Steve Heim, Matthew Millard, Charlotte Le Mouel, Alexander Badri-Spröwitz

**Affiliations:** 1 Intelligent Control Systems Group, Max Planck Institute for Intelligent Systems, 70569 Stuttgart, Germany; 2 Optimization, Robotics and Biomechanics, Institute of Computer Engineering, University of Heidelberg, 69120 Heidelberg, Germany; 3 Department of Movement Science, Institute of Sport and Exercise Sciences, University of Münster, 48149 Münster, Germany; 4 Dynamic Locomotion Group, Max Planck Institute for Intelligent Systems, Stuttgart, Germany

**Keywords:** muscle, damping, stability, locomotion, slip

## Abstract

It is currently unclear if damping plays a functional role in legged locomotion, and simple models often do not include damping terms. We present a new model with a damping term that is isolated from other parameters: that is, the damping term can be adjusted without retuning other model parameters for nominal motion. We systematically compare how increased damping affects stability in the face of unexpected ground-height perturbations. Unlike most studies, we focus on task-level stability: instead of observing whether trajectories converge towards a nominal limit-cycle, we quantify the ability to avoid falls using a recently developed mathematical measure. This measure allows trajectories to be compared quantitatively instead of only being separated into a binary classification of ‘stable' or ‘unstable'. Our simulation study shows that increased damping contributes significantly to task-level stability; however, this benefit quickly plateaus after only a small amount of damping. These results suggest that the low intrinsic damping values observed experimentally may have stability benefits and are not simply minimized for energetic reasons. All Python code and data needed to generate our results are available open source.

## 1. Introduction

Compliance is a defining characteristic of running motion [1], and models of running often include idealized springs. One of the most popular models is the spring-loaded inverted pendulum (SLIP) model [2]. Despite its simplicity, its parameters can be fit to closely emulate the ground reaction forces and centre-of-mass (CoM) kinematics of steady running in humans [3] and other animals [4]. Daley & Biewener [5] used the SLIP model to explain how well birds could run over an unexpected step-down perturbation. Guinea fowl were habituated to run over a level runway, after which a pothole camouflaged with tissue paper was introduced. Despite the unexpected perturbation, the birds did not stumble and fall. Because the duration of the step in the pothole was so short, the researchers concluded that the birds remained in open-loop control: they did not react or re-plan, but executed the step as if still running on level ground.

Even though damping effects are often observed in animal movement [6–8], they are only seldomly included in models. Birn-Jeffery *et al*. [9] included damping in a modified SLIP model and found that this leads to more accurate predictions of ground reaction forces in running birds. Similar observations have also been made with other damped models [10].

Damping elements are sometimes added to SLIP-based models to study energy-injecting controllers [11–13]. These studies, however, focus on the stabilizing effects of the presented controllers and their potential applications in robotics. Shen & Seipel [14] studied the passive stability of a SLIP-like model with a viscous damper placed in parallel to the spring and driven by a constant hip-torque during stance to compensate for energy dissipation. They found that increasing damping and hip-torque tends to improve the stability of their model, though not at very small values of damping. This is likely owing to the unrealistic non-zero damping force at touch-down, which they addressed in a subsequent model [10]. Their study analyses the model's open-loop stability, which may not capture important aspects of animal motion, as we discuss below.

Classical definitions of stability are based on the analysis of equilibrium points or limit-cycles and hinge on the notion of convergence. An equilibrium point (limit-cycle) is stable if nearby points (orbits) eventually converge towards it, and unstable if they diverge. Locomotion is often thought of as a limit-cycle, which allows a wealth of mathematical tools to be used for analysis [15–19]. However, Birn-Jeffery *et al*. [9] suggest that convergence may not be a task-level priority for running birds. Moore *et al*. [20] suggest that animals may even choose to prioritize unsteady motion to thwart would-be predators and also observe that bipedal Jerboas often switch between different gaits for the same speed. Humans are observed to show significant step-to-step variability even when moving at a fixed speed on a treadmill [21–23].

We define task-level stability as the ability to avoid falling. This definition of stability is less restrictive than definitions that require convergence and does not conflict with other task-level priorities. We show the influence that damping has on how robustly a model can maintain task-level stability. To quantify this robustness, we use a *measure of viability*, developed in Heim *et al*. [24] and presented below.

## 2. Materials and methods

### (a) Measure of viability

Intuitively, the measure of viability quantifies how easy it is to avoid ever failing, which in our context corresponds to the body hitting the ground, i.e. falling.

A system is said to be in an *un*viable state if it is impossible to avoid falling within finite time, regardless of the control inputs chosen [25]. If the state is viable, on the other hand, there must exist at least one control input that takes the system to another viable state, and falling can be avoided forever by continuing to apply a viability-maintaining control input.

Because of this recursive property, viability can be evaluated on a single step, and heuristics such as steps-to-falling can be avoided [12,16]. Since viability only requires the ability to avoid falling, it does not impose strong requirements such as limit-cycle convergence [3,10,17,26].

**Figure 1.**
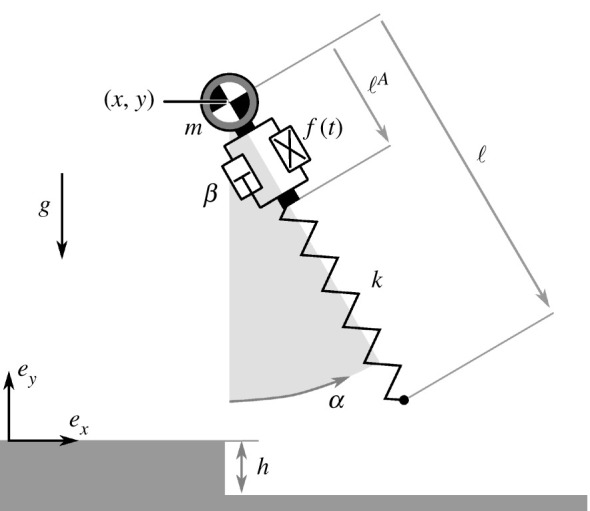
The DASLIP model extends the classical SLIP model with a damper-actuator module.

The measure of viability is the *n*-dimensional volume of control inputs that keep the model viable. In our model, shown in figure 1, the leg angle-of-attack during flight is the only active control input; the measure is, therefore, the range of angles that keep the state viable. If the measure is zero, the state is unviable and the system will inevitably fall within a finite number of steps, no matter which angle is chosen. The greater the measure, the more robust the model is to imprecise control, regardless of whether this imprecision is owing to noisy motor-control, perturbations, or other causes [27–30]. Indeed, if the measure is too small, the model may not be able to robustly avoid falling, even if it is theoretically possible.

For a given system, we can pre-compute this measure for every state with an iterative algorithm. For nonlinear systems, the computational cost of this algorithm currently scales exponentially with the number of states and control inputs, and studies are therefore limited to low-dimensional models. The results presented here require roughly a full day of computation on a 24-core desktop. For mathematical and algorithmic details, see [24,31]. The Python code for computing this measure and all results in this paper is available in the electronic supplementary material [32] and online at github.com/sheim/vibly.

### (b) Experiment

Inspired by the guinea fowl experiments of Daley and Biewener [5], we test for the ability to avoid falling after an unexpected ground-height perturbation. We start by finding a nominal limit-cycle to represent running over level ground. Then, we repeatedly simulate a single step from one flight apex to the next, keeping the damping coefficient and initial states fixed while sweeping through a range of ground-height perturbations. Since the ground-height perturbation is assumed to be unexpected, all control inputs are applied as if running on level ground. We then evaluate the pre-computed measure of viability at the apex state after the perturbed step and use this measure to compare different trajectories.

This battery of simulations is repeated for a range of damping values. Finally, we compare the viability measure after the perturbation, but with different amounts of damping.

### (c) Model

We refer to our model, shown in figure 1, as the DASLIP model since it extends the SLIP model with a damper-actuator module.

As in the standard SLIP model, a point mass with coordinates (*x*, *y*) and mass *m* represents the body, and a massless leg with length ℓ represents the leg. During flight, the leg is held at a constant angle-of-attack *α*, and the motion of the point mass is only affected by gravity. During stance, it is additionally affected by the leg force *F*_leg_. The equations of motion of the CoM are given by:

$$\begin{array}{rl} x & =-\frac{F_{leg}}{m}\text{sin}\alpha \\ \text{and}\;y & =\frac{F_{leg}}{m}\text{cos}\alpha -mg. \end{array}$$

In the SLIP model, the massless leg is composed of a spring with resting length ℓ*_k_* and stiffness *k*, such that the leg force is determined solely by the spring compression: $F_{leg;SLIP}=k(\ell -\ell_{k})$. In our model, the system state is extended with the length *ℓ^A^* of the damper-actuator module. This module is placed in series with the spring, such that:

$$F_{\mathrm{leg}}=k(\ell -\ell^{A}-\ell_{k}).$$

This module is composed of a time-dependent force source *f*(*t*) in parallel to a viscous damper with coefficient *β*, such that the total force of the module, *F_DA_*, is given by:

$$F_{DA}=f(t)-\beta {\dot{\ell }}^{A}.$$

The dynamics of ℓ*^A^* are found by resolving the force balance between the module and the spring, $F_{DA}=F_{leg}$:

$${\dot{\ell }}^{A}=\frac{\;f(t)-F_{\mathrm{leg}}}{\beta }.$$

We set the time-dependent force source *f*(*t*) such that the DASLIP model exactly follows the nominal limit-cycle of a SLIP model with the same initial states and parameters. This is achieved by matching *f*(*t*) to the force profile of the SLIP model. In the absence of perturbations, *f*(*t*) holds the module length ℓ*^A^* fixed, and the damper has no effect. When encountering a perturbation in ground-height *h*, the spring force will no longer be matched by *f*(*t*), the module length ℓ*^A^* will change, and the damper will counteract this displacement.

The damping coefficient *β* must be greater than zero, but it can be set arbitrarily small. With infinitesimal damping, the total leg force will approach that of the open-loop actuator. By setting an infinitely high damping coefficient, the damper will resist all movement of the damper-actuator module, locking it in place. In this limit, the model behaves exactly like the SLIP model, also when perturbed.

While the SLIP model subsumes the entire leg behaviour into a massless spring, the DASLIP abstracts the muscles and tendons separately as the damper-actuator module and the spring, respectively. The damper-actuator module has two important properties of muscle: it develops little force unless activated and it has intrinsic damping [6]. We have modelled tendon as an idealized spring [33] in series with the damper-actuator module to mimic the topology of biological muscle and tendon.

For the simulations described above, we assume open-loop running during the perturbed step, and therefore no active control inputs. To pre-compute the viability measure, however, we need to define the control inputs that the model may choose in subsequent recovery steps. We define as control input the angle of attack *α*, which can be instantaneously reset during the flight phase. Both experimental observations [5] and model simulations [26,34] indicate *α* has an important influence on the CoM motion, making it a natural choice.

For the sake of computational tractability, we do not include additional control inputs to modulate *f*(*t*), though this would arguably also be a realistic control input available to the bird. Instead, we automatically shift the profile of *f*(*t*) so it always coincides with touch-down, which is determined by the control input *α*. In other words, we use a single control input to determine both the leg angle and muscle activation. The validity of this modelling choice is sensitive to the nominal limit-cycle, since this determines the shape and duration of *f*(*t*). In our experiments, this shift results in a reasonable force profile for the controlled recovery steps.

### (d) Choice of parameters

For the nominal limit-cycle, we use recorded data of a guinea fowl running over level ground, which was kindly provided by Monica Daley from a previous study [35]. We use averages of all steps of a single trial over level ground. We define the virtual leg length ℓ_0_ as the distance between the bird's CoM and foot at touch-down. This length is used to determine the spring resting length *ℓ_k_* and initial muscle length *ℓ^A^* at a 9 : 1 ratio, and to normalize results. We also use these averages to determine the remaining initial and all remaining parameters except for spring stiffness. We then numerically fit a spring stiffness *k* that produces limit-cycle motion for an equivalent SLIP model.

For the results shown, we use the parameters in table 1.

**Table 1.** Parameters.

name	symbol	value	normalized value
parameters			
mass	*m*	1.37 kg	
spring resting length	ℓ*_k_*	19.4 cm	0.9 ℓ_0_
spring stiffness	*k*	840.4 N m^−1^	13.6 mg/ℓ_0_
landing angle of attack	*α*	34.1°	
states			
height at apex	*y*_0_	19.6 cm	0.9 ℓ_0_
velocity at apex	${\dot{x}}_{0}$	2.7 m s^−1^	12.3 ℓ_0_/*s*
damper-actuator module length	ℓ*^A^*	2.2 cm	0.1 ℓ_0_

## 3. Results

**Figure 2.**
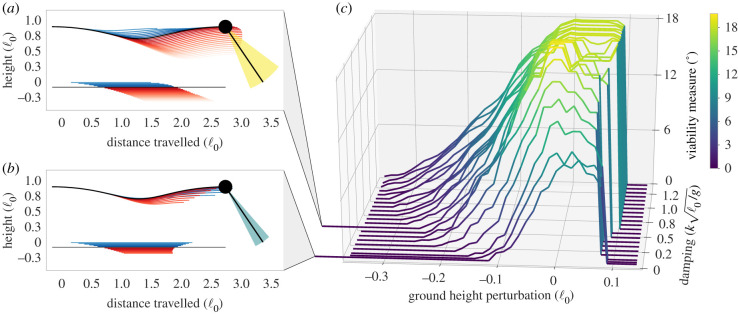
(*a*,*b*) The trajectories for two specific damping values, over a range of ground-height perturbations: (*a*) $0.498k\sqrt{\ell_{0}/g}$ and (*b*) $0.007k\sqrt{\ell_{0}/g}$. The limit-cycle trajectories over level ground are coloured in black. Trajectories for step-up and step-down perturbations are coloured blue and red, respectively. The ground-height for perturbations is also coloured starting from the point-mass position at touch-down until take-off. For clarity, unviable trajectories are not visualized. At the end of the step, we also visualize the viability-maintaining control inputs for the nominal limit-cycle, colourized according to the viability measure. In (*c*) we visualize the viability measure (vertical axis) at the apex reached after each ground-height perturbation (horizontal axis), where each line corresponds to a specific damping value *β*.

In figure 2, we visualize the simulated trajectories for two specific values of damping: in (i) $\;\beta =0.498k\sqrt{\ell_{0}/g}=58.8\;\;\mathrm{Ns}\;{\text{m}}^{-1}$ and in (ii)$\;\beta =0.007k\sqrt{\ell_{0}/g}=0.8\;\;\text{Ns}\;{\text{m}}^{-1}$. In figure 2*c*, we visualize the viability measure for all simulations: each line corresponds to a battery of ground-height perturbations for a specific damping value *β*.

At the right edge of figure 2*c*, the apparent cliff in viability measure is owing to the model stumbling on the raised ground. On the left side, the maximum step-down perturbation from which recovery is possible is smaller for lower damping values. More importantly, the overall viability measure is low, even for the nominal limit-cycle. As the damping value is increased from 0.007 to $0.498k\sqrt{\ell_{0}/g}$, the range of recoverable step-down perturbations roughly doubles from 9% ℓ_0_ = 2.0 cm to 21% ℓ_0_ = 4.5 cm. More importantly, the viability measure also increases substantially, for the nominal limit-cycle trajectory, from 9.4° to 19.5°.

Further increasing damping continues to increase the largest step-down perturbation. However, the increase in viability measure slows down rapidly. Indeed, when increasing the damping value up to $1.35\;k\sqrt{\ell_{0}/g}=159.7\;\text{Ns}\;{\text{m}}^{-1}$, the maximum viability measure saturates at 18°, and the range of perturbations that reach this maximum, the ‘plateau’ in figure 2*c*, only increases marginally.

## 4. Discussion

The DASLIP model extends the classical SLIP model with a damper-actuator module in the leg, as an abstract representation of muscle activation and damping. The damping coefficient allows us to smoothly blend between two different leg models: a feed-forward force source when damping tends to zero and an idealized spring when damping tends to infinity. For limit-cycle running on level ground, these two models (and all combinations) behave in the same way owing to the choice of the time-dependent force source *f*(*t*).

Faced with unexpected ground-height perturbations, our model showed poor robustness when minimally damped. Increasing the damping coefficient increases both the viability measure and the range of perturbations that can be negotiated. However, the maximum viability measure quickly reaches a plateau, and further increasing damping only widens this plateau marginally. In other words, the benefit in robustness owing to increasing damping is limited.

Robustness is important because motor-control is inherently noisy and imprecise [27–30]. In studies of running guinea fowl and pheasants [35,36], the birds exhibited notable variability in their control of the landing angle-of-attack, with the standard deviation typically ranging between 3° and 8° depending on the presence and size of (visible) obstacles. In the context of our results, this would suggest that only the upper half of figure 2*c* represents perturbations that can be negotiated robustly: wherever the measure is small compared to the control input variability, recovery is possible, but not robust.

We believe a quantitative study to identify the minimum threshold for the viability measure (how ‘far down the mountain' are animals willing to go?) would be of great interest, albeit challenging. In particular, it is not trivial to assess if motor-control variability is owing to a limit in capability, or simply because greater precision is not needed for a given task. Such a study would provide a further tool with which to compare task-level priorities [35] and, in particular, to study behaviour involving risk [37,38].

Damping is not free of cost, since it will also reduce muscle efficiency. In our model, the plateau in improved robustness can be reached with only a small cost in energy efficiency, which would likely be negligible for most animals. It would be interesting to study if this trade-off becomes relevant in specialized animals. In particular, if task-level stability is not a concern, but there is a strong evolutionary pressure to optimize efficiency, it may still be beneficial to further minimize damping. This may be the case in desert habitats, where the environment is relatively flat and does not require agility [39], and where falls do not determine predator–prey interactions [20,40].

These results may also be informative for robot design. There are few examples of legged robots that incorporate physical dampers in their design [41–43]. These efforts have, however, not focused on task-level stability, and the potential benefit of damping in this context remains to be explored.

## Supplementary Material

Figure 2.a) complete

## Supplementary Material

Figure 2.b) complete

## Data accessibility

All data and code for this submission are available online at https://github.com/sheim/vibly. A slimmed-down version of the repository (including only code and data relevant to the submission) has been uploaded to Dryad for permanence: https://doi.org/10.5061/dryad.44j0zpcbj.
